# Inequities in COVID-19 Omicron infections and hospitalisations for Māori and Pacific people in Te Manawa Taki Midland region, New Zealand

**DOI:** 10.1017/S0950268823000572

**Published:** 2023-04-24

**Authors:** Jesse Whitehead, Han Gan, Jacob Heerikhuisen, George Gray, Trevor Richardson, Paul Brown, Ross Lawrenson

**Affiliations:** 1 Te Ngira Institute for Population Research, University of Waikato, Hamilton, New Zealand; 2 School of Computing and Mathematical Sciences, University of Waikato, Hamilton, New Zealand; 3 Te Whatu Ora Health New Zealand – Bay of Plenty (Previously Bay of Plenty District Health Board), Tauranga, New Zealand; 4 Waikato Medical Research Centre, Te Huataki Hauora School of Health, University of Waikato, Hamilton, New Zealand; 5 Te Whatu Ora Health New Zealand – Waikato (Previously Waikato District Health Board), Hamilton, New Zealand

**Keywords:** COVID-19, health inequalities, hospitalisations, Māori, Pacific, vaccination

## Abstract

COVID-19 impacts population health equity. While mRNA vaccines protect against serious illness and death, little New Zealand (NZ) data exist about the impact of Omicron – and the effectiveness of vaccination – on different population groups. We aim to examine the impact of Omicron on Māori, Pacific, and Other ethnicities and how this interacts with age and vaccination status in the Te Manawa Taki Midland region of NZ. Daily COVID-19 infection and hospitalisation rates (1 February 2022 to 29 June 2022) were calculated for Māori, Pacific, and Other ethnicities for six age bands. A multivariate logistic regression model quantified the effects of ethnicity, age, and vaccination on hospitalisation rates. Per-capita Omicron cases were highest and occurred earliest among Pacific (9 per 1,000) and Māori (5 per 1,000) people and were highest among 12–24-year-olds (7 per 1,000). Hospitalisation was significantly more likely for Māori people (odds ratio (OR) = 2.03), Pacific people (OR = 1.75), over 75-year-olds (OR = 39.22), and unvaccinated people (OR = 4.64). Length of hospitalisation is strongly related to age. COVID-19 vaccination reduces hospitalisations for older individuals and Māori and Pacific populations. Omicron inequitably impacted Māori and Pacific people through higher per-capita infection and hospitalisation rates. Older people are more likely to be hospitalised and for longer.

## What is already known on this topic?

Ethnic differences in the impact of COVID-19 have been widely reported. In NZ, Māori and Pacific people experienced a disproportionate health burden from the Delta variant of COVID-19. This mirrors historic experiences of pandemics such as the 2009 influenza A (H1N1) pandemic and 1918 influenza pandemic. Globally, older people are at highest risk of serious illness and death from COVID-19 infection. mRNA vaccines provide good protection against serious illness and death from the Omicron variant of COVID-19.

## What this study adds?

Māori and Pacific people in the Te Manawa Taki Midland region of NZ were inequitably impacted by the Omicron wave of COVID-19. Per-capita infections among Māori and Pacific people were higher, and peaked earlier than for people of Other ethnicities. While infection rates were also higher among younger people, the risk of hospitalisation was highest, and the length of hospitalisation longest, for the 75+ age group. While three doses of the Pfizer mRNA vaccine provide good protection against hospitalisation for COVID-19, age and ethnic differences in hospitalisation rates persist across vaccination status.

## How this study might affect research, practice, or policy?

This research provides evidence indicating that Māori, Pacific, and older people should be prioritised in vaccination programmes and infectious disease control measures. This study also highlights the changing impact of an infectious disease outbreak over time, where per-capita infections were initially highest among Māori and Pacific and younger people, before a transition to higher rates among Other ethnicities and the elderly. This indicates that more targeted approaches to different population groups may be required at different stages of infectious disease outbreaks.

## Background

COVID-19 has impacted the health of populations around the world, with sickness, hospitalisation, and death [1]. Inequities in hospitalisations and death occur across multiple contexts by ethnicity, socioeconomic status, and Indigenous identity, with the risk of hospitalisation and death substantially increasing with age and comorbidities [2–8]. The original strain was particularly devastating due to the unavailability of vaccines to protect against serious illness and death. While high levels of hospitalisation and mortality continue to be observed with subsequent variants [9], mRNA vaccine boosters provide a high level of protection against hospitalisation and death, despite Omicron’s ‘vaccine-escape’ properties [10]. However, ethnic differences in vaccine effectiveness for Omicron have not been examined. Ethnic inequities in the impact of COVID-19 are shaped by structural racism and other power structures, and arise through differential: exposure to the virus; vulnerability to infection; disease consequences; and effectiveness of control measures [11].

Relatively little data from Aotearoa New Zealand (NZ hereafter) exist around the impact of COVID-19, and the effectiveness of the Pfizer vaccine in preventing serious illness, in different population groups, particularly for the Omicron wave. Early data indicated that Māori and Pacific people were, respectively, 2.5 and 3 times more likely to become hospitalised during the first wave of COVID-19 in NZ, even when controlling for age and underlying conditions [12]. The health burden of the Delta outbreak in NZ was disproportionately experienced by Māori and Pacific people [13]. While it was projected that an Omicron outbreak could result in between 8,000 and 33,040 hospitalisations [14], the likely impact on Māori and Pacific people and specific age groups was not explored.

Health inequities in NZ, particularly for Indigenous Māori and Pacific people, are well documented [15–20]. These include high levels of severe outcomes from previous infectious disease outbreaks such as the 2009 influenza A (H1N1) pandemic and the 1918 influenza pandemic [21, 22]. Inequities are driven by our history of colonisation and contemporary racism in the health system and wider society, and shaped by differential access to the social determinants of health such as poorer access to housing and quality healthcare [23–29].

Older Māori and Pacific people, the elderly, and people with underlying health conditions had earlier access to COVID-19 vaccines [30]. However, the younger age structure of Māori and Pacific populations in NZ [31] meant that many were not eligible for vaccination until later in the age-based rollout. Furthermore, ongoing structural and accessibility barriers have meant that overall uptake of COVID-19 vaccinations has been delayed and lower among Māori and Pacific people [32–34]. Effective public health measures during 2020 meant that NZ largely eliminated COVID-19 and avoided a significant wave of cases [35]. However, with the Delta variant, NZ moved to a suppression strategy in the latter half of 2021 with a reliance on vaccination to limit serious illness and death. The Omicron outbreak began in 2022, and by 31 January, 94% of the eligible NZ population had completed two Pfizer doses, whereas 67% had received a third dose [36]. Ethnic differences in uptake persisted, with 85% of eligible Māori vaccinated with two doses.

To forecast the impact of Omicron on local health services, we worked with four district health boards (DHBs) from the Te Manawa Taki region (Waikato, Bay of Plenty, Taranaki, and Tairāwhiti) and Hawke’s Bay DHB (see Supplementary Figure 1) serving a population of 1,074,500 individuals, of which 281,000 (26%) were identified as Māori and 29,000 (2.7%) as Pacific [37, 38], with substantially younger age structures (see Table 1). This study aims to examine the impact of the Omicron outbreak on Māori, Pacific, and people of Other ethnicities and how this interacts with age and vaccination status. We investigate differences in virus transmission over time by age and ethnicity and quantify the risk of hospitalisation by age, ethnicity, and vaccination status, before exploring differences in length of hospitalisation.

**Table 1.** Estimated resident population of the study region by age and ethnicity

Age band	Māori	Pacific	Other
0–11	69,450	6,494	95,456
12–24	63,379	6,656	102,966
25–44	71,530	8,748	188,223
45–64	54,206	5,210	207,883
65–74	13,672	1,151	95,976
75+	6,443	579	76,480

## Methods

### Data

The primary denominator is the study area population, disaggregated by age, ethnicity, and DHB region. This comes from two customised Stats NZ datasets. First, the Estimated Resident Population (year ending June 2021) disaggregated by age, sex, and geographic location [37]. Population estimates by ethnicity are unavailable for intercensal years, so prioritised ethnicity was apportioned from a second customised dataset of the Estimated Resident Population for the year ending June 2018 [38]. Vaccination status was provided by each DHB’s COVID-19 immunisation registers, and included data on age, ethnicity, and geographic location. Vaccination status was categorised, at the time of infection, as (a) unvaccinated (zero-to-one Pfizer dose), (b) fully vaccinated (two doses), or (c) boosted (three or more doses). Children of 0–4 years of age are unable to receive any doses of COVID-19 vaccines, whereas booster vaccinations are only available to those aged 16 years and older. Numerator data on incident cases of COVID-19 were provided by each DHB, and included date of recorded infection, age, prioritised ethnicity, geographic location, and vaccination status of the individual. The study period coincides with a transition from cases being confirmed by a polymerase chain reaction (PCR) test administered at COVID-19 testing centres and analysed in laboratories, to a ‘self-testing’ regime using rapid antigen tests (RAT) in mid-to-late February 2022. RATs were made available free of charge at locations that previously provided PCR tests. RATs were also delivered to more remote communities and were available to purchase from places such as supermarkets and pharmacies. Test results were reported to the Ministry of Health, either online or via telephone. While a positive test result meant that individuals and the people they lived with were legally required to self-isolate, which may have been burdensome, it also meant that they were eligible for free primary health care and additional social support from the DHB to support their self-isolation. COVID-19-related hospitalisation numerator data were also provided weekly and disaggregated by date and time of hospitalisation, age, gender, prioritised ethnicity, and vaccination status. COVID-19 hospitalisations were defined as hospital admissions where the patient was a confirmed COVID-19 case (ICD-10 AM code: U07.1). This includes patients where COVID-19 was the primary cause of hospitalisation, and patients hospitalised for other reasons who presented *with* COVID-19 infections.

### Infection and hospitalisation rates

Using the above data, daily per-capita infection rates by age and ethnicity were calculated for the period between 1 February to 29 June 2022. The 7-day rolling average infection rate was calculated to smooth daily differences in reporting by averaging the infection rate across the previous 7 days. Hospitalisation rates were calculated for the same study period using DHB-provided incident cases as the denominator and hospitalisations as the numerator. Differential reporting of COVID-19 infections may influence hospitalisation rates, and therefore daily hospitalisation rates were also calculated using census population data as the denominator. Length of hospitalisation was calculated as the difference in date and time of admission and discharge for all reported hospitalisations.

### Statistical analysis

Using confirmed positive case and hospitalisation data from DHBs, unadjusted hospitalisation rates for both reported infections and the population, stratified by ethnicity, age, and vaccination status, were calculated. Tairāwhiti DHB was not included due to a lack of available data. To quantify the effects of ethnicity, age, and vaccination, we fit a multivariate logistic regression model in R. We initially fit the logistic regression model using all main effects and first-order interaction terms. Then, using Akaike Information Criterion (AIC) as a model selection procedure to choose an optimal subset of explanatory variables, the final model included vaccination status, ethnicity, and age as main effects, and interaction terms of *age* × *ethnicity* and *age* × *vaccination status.* ORs that estimate the effect of one factor averaged across all combinations of the remaining factors were calculated using the *emmeans* package in R. Finally, hospitalisation length of stay was examined using a linear model with ethnicity, age, and vaccination status as main effect terms.

### Ethics

Ethical approval was provided by the University of Waikato Human Research Ethics Committee (HREC 2022-50).

## Results

### Infection rates

**Figure 1.**
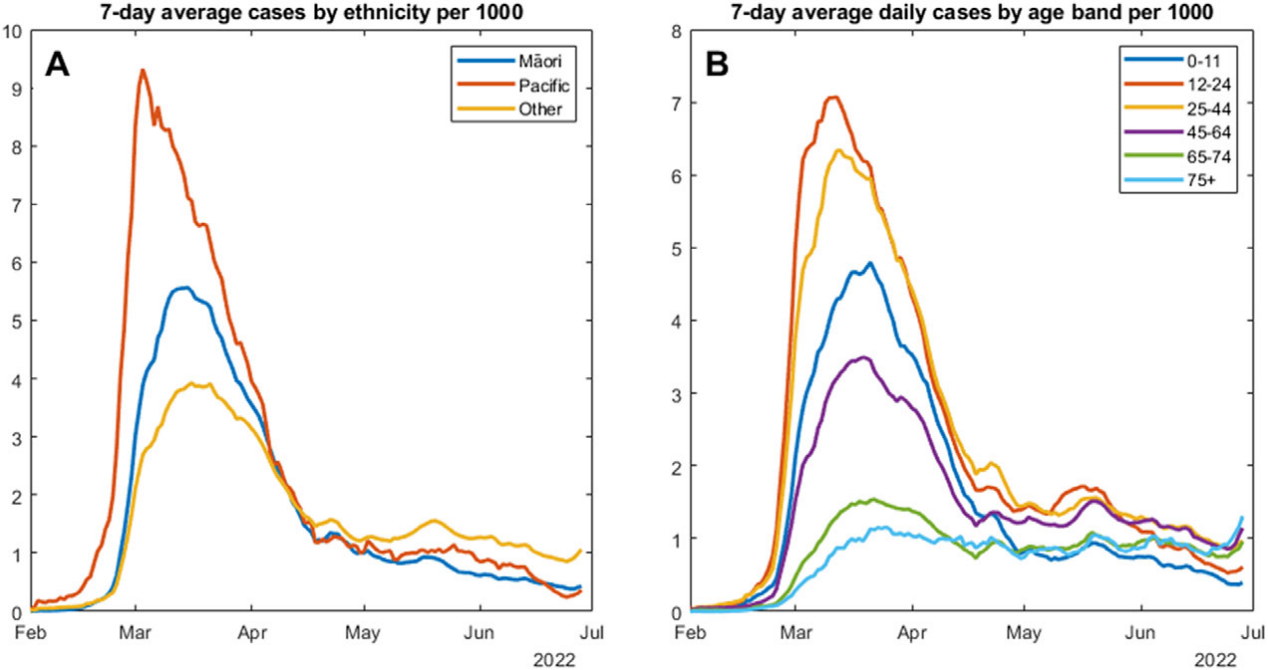
Seven-day average confirmed COVID-19 cases per 1,000 individuals by ethnicity (a) and age group (b).

Figure 1a indicates a rapid rise in the overall infection rate within the study region, starting in early February and peaking at 4.5 cases per 1,000 in mid-March. The reported per-capita infection rate in Māori and Pacific people peaked earlier and was much higher than the rate for non-Māori, non-Pacific people of ‘Other’ ethnicities. The peak, occurring in late February, was almost three times greater in Pacific people at over 9 per 1,000. For Māori, the peak infection rate of over 5 per 1,000 occurred in early March. There were also major differences in the spread of COVID-19 infections between age groups. Figure 1b shows that the highest per-capita rate of infection, occurring earliest in the outbreak, was among the 12–24 age group, closely followed by the 25–44 age group. Infections among children in the primary and pre-school age group (0–11 years) peaked slightly later and at a lower rate. The infection rates in the oldest two age groups (65–74 and 75+) had a less dramatic curve, and although they peaked in mid-to-late March, they have not risen above 2 per 1,000. Of concern, however, is the uptick in reported infections in the 75+ age group, which began in late June 2022. By the end of the study period, this oldest age group had the highest per-capita infection rate. Supplementary Figure 2 presents infection rates by ethnicity for 0–44-year-olds. Infection rates are higher in younger Pacific people, but similar to Māori and ‘Other’, suggesting that high overall Māori infection rates may be influenced by the younger Māori age structure.

### Hospitalisation

Table 2 presents modelled per-capita hospitalisation rates, indicating that these are influenced substantially by age, ethnicity, and vaccination status. Rates of hospitalisation are highest for unvaccinated individuals in all age bands and for each of the three ethnic groups. Hospitalisation rates are also higher for Māori and Pacific people, compared to Other ethnicities, for all age bands, and regardless of vaccination status. Among unvaccinated children aged 0–11, Māori and Pacific people are 1.9 and 2.1 times more likely to be hospitalised than Other ethnicities. This pattern of inequitable hospitalisation rates persists in the boosted population aged 75 years and older, where Māori and Pacific people are 1.4 and 1.2 times more likely to be hospitalised than Other ethnicities.

**Table 2.** Fitted multivariate logistic regression model hospitalisation cases per 1,000 confirmed infections, and per 1,000 people by ethnicity, age band, and vaccination status for the Bay of Plenty, Taranaki, Hawke’s Bay, and Waikato district health board regions combined from 1 February to 29 June 2022

		Total hospitalisations			Modelled hospitalisations per 1,000 confirmed infections			Modelled hospitalisations per 1,000 people		
Vaccination status	Age band	Māori	Pacific	Other	Māori	Pacific	Other	Māori	Pacific	Other
Unvaccinated	0–11	200	24	250	21	23	11	4	6	2
	12–24	71	1	34	26	12	11	5	8	3
	25–44	124	12	95	51	48	28	7	11	5
	45–64	83	12	64	107	110	43	6	11	4
	65–74	43	4	45	261	245	137	16	27	11
	75+	29	3	97	411	377	322	21	36	15
Two doses	0–11	9	2	6	9	9	5	–^a^	–^a^	–^a^
	12–24	77	10	74	6	3	3	2	3	1
	25–44	160	26	190	13	12	7	5	6	2
	45–64	104	15	109	27	28	10	6	7	3
	65–74	33	4	88	134	125	65	19	21	7
	75+	47	4	126	269	242	201	53	60	22
Three doses	0–11	N/A	N/A	N/A	N/A	N/A	N/A	N/A	N/A	N/A
	12–24	20	0	37	11	5	5	5	4	2
	25–44	82	19	178	11	10	6	6	5	2
	45–64	127	19	227	19	19	7	5	4	2
	65–74	91	8	240	53	49	25	9	8	4
	75+	68	10	725	130	115	93	28	23	11

a NB: Due to small number issues modelled per 1,000 people, hospitalisation rates are not presented for the 0–11 ‘two-dose’ group. Supplementary Table 2 includes full raw hospitalisation rates using both denominators.

ORs of hospitalisation for ethnicity, vaccination status, and age bands are presented in Table 3. All ORs are multivariate results. For example, the OR estimate for Māori represents the hospitalisation odds for Māori compared to Other averaged over all age bands and all vaccination statuses. Using the fitted multivariate logistic regression model, we estimate that the hospitalisation risk is, respectively, 2.032 and 1.745 times higher for Māori and Pacific people compared with Other ethnicities. While 0–11-year-olds are excluded from vaccination status and age-group ORs due to the Ministry of Health age-eligibility criteria, vaccination status and age also appeared to be highly associated with hospitalisation rates.

**Table 3.** Odds ratios of modelled hospitalisation risk based upon hospitalisation risk per confirmed infection from 1 February to 29 June 2022, by ethnicity, vaccination status, and age group for the Bay of Plenty, Taranaki, Hawke’s Bay, and Waikato district health board regions combined.

	Odds ratio estimate	95% confidence interval
Māori/other	2.03	(1.86, 2.22)
Pacific/other	1.75	(1.40, 2.19)
Māori/Pacific	1.16	(0.93, 1.46)
Unvaccinated/two doses	3.13	(2.75, 3.56)
Unvaccinated/three doses	4.64	(4.08, 5.28)
Two doses/three doses	1.48	(1.33, 1.66)
25–44/12–24	2.21	(1.59, 3.08)
45–64/12–24	4.01	(2.85, 5.64)
65–74/12–24	14.97	(10.01, 22.37)
75+/12–24	39.22	(26.04, 58.82)

The interaction terms between age and ethnicity suggest that the differences in ethnicity vary by age, with distinct ethnic differences in hospitalisation rates for <75-year-olds that are less noticeable in the 75+ age group. The interaction terms between age and vaccination status also suggest that the impact of vaccination varies between age bands. In the younger age groups, three vaccination doses reduced the risk of hospitalisation by approximately 50%, whereas for those aged 75+, three vaccination doses appear to have a greater impact and reduce hospitalisations by approximately 70%.

To test whether potential ethnic differences in reporting of COVID-19 infections may have influenced the pattern of higher hospitalisation rates among Māori and Pacific people, the census-based denominator was used to calculate per-capita hospitalisations by ethnicity (see Supplementary Figure 3). The same pattern of higher hospitalisation rates among Māori and Pacific people is observed particularly during the early parts of the Omicron outbreak, suggesting that any differential reporting of COVID-19 infections is unlikely, or has not substantially impacted the modelled hospitalisation rates presented in Table 2. Supplementary Table 2 displays the raw hospitalisation rates using both denominators for the entire study period and shows that the pattern of higher hospitalisation rates for Māori and Pacific people persists when the census-based denominator is used.

### Length of hospitalisation

Fitting a linear model with main effects of age, ethnicity, and vaccination status, we find substantial differences in length of hospital stay by age. Relatively minimal differences are noted by ethnicity and vaccination status, suggesting that once a patient is admitted to hospital, age is the key factor determining length of stay (see Supplementary Table 3).

## Discussion

### Statement of principal findings

Our analysis indicates important population differences in experiences of the Omicron COVID-19 wave in the Te Manawa Taki and Hawke’s Bay regions. Reported per-capita cases of Omicron were highest and occurred earliest among Pacific and Māori people. Reported cases were also high among the three youngest age groups, and highest among 12–24-year-olds. While per-capita cases among those aged 75+ were mostly relatively low, by July 2022, this age group had the highest reported infection rate. Substantial and statistically significant differences in hospitalisation rates were observed by ethnicity, age, and vaccination status. Māori and Pacific people were more likely to be hospitalised than Other ethnicities, and the hospitalisation risk for elderly people was 39.2 times higher than those aged 12–24. Hospitalisation risk for unvaccinated people was more than four times higher than those who received three doses. Our data suggest that hospitalisation length is strongly related to age, with no statistically significant differences by ethnic group or vaccination status. The average length of stay for people aged 75+ was approximately five times longer than that for children aged 11 years and younger.

### Meaning of the study: possible mechanisms and implications for clinicians or policymakers

Our research provides evidence of the effectiveness of COVID-19 vaccination and booster shots in reducing hospitalisations, for all ethnic groups and particularly for older individuals. Among those aged 75 years and older, two and three vaccination doses resulted in three-to-four times less likelihood of hospital admission compared with unvaccinated people. The observed inequity in COVID-19 infections for Māori and Pacific people, with higher rates of infection that occurred earlier in the outbreak, reinforces previous findings of higher health burdens for Māori and Pacific people from the 2009 H1N1 pandemic [22], and suggests that these populations may again experience higher infection rates during future outbreaks of COVID-19, or other pandemics. The observed ethnic inequities in hospitalisation rates at all age groups, and for all vaccination statuses, highlight the importance of preferential prioritisation of Māori and Pacific populations in vaccination programmes. Preferential targeting through prioritisation of Māori and Pacific people and meaningful collaboration with iwi (the Māori word *iwi* refers to ‘extended kinship group, tribe, nation, people, nationality, and race – often refers to a large group of people descended from a common ancestor and associated with a distinct territory’ (https://maoridictionary.co.nz)) and Pacific communities is needed to achieve equitable protection against future pandemics. This could include the removal of any age sequencing for priority populations and geographic targeting of Māori and Pacific populations to improve vaccine access for communities at risk of worse outcomes [32, 39]. COVID-19 infections have the greatest impact on the elderly with high rates of hospitalisation and longer lengths of stay even in those who have been fully vaccinated or received booster doses. This emphasises the importance of continuing to protect the older age groups from infection through public health measures such as mask wearing and the isolation of people infected with COVID-19. These findings also provide evidence for the differential risk of exposure to COVID-19 and vulnerability to infection [11] of Māori, Pacific, and younger people at the start of the 2022 Omicron wave, and the transition to greater risk of exposure to the consequences of infection for older people in June and July 2022. It highlights the differential disease consequences, particularly for older, Māori, and Pacific people who were more likely to be hospitalised. This emphasises the importance of three vaccination doses as part of a suite of control measures to reduce the inequitable impact of COVID-19.

### Strengths and weaknesses of the study

To the best of our knowledge, this study is the first in the NZ context to examine the unequal impact of the Omicron wave on different population groups, with a specific focus on Māori and Pacific priority populations and vaccination status. Our time-series analysis highlights the changing impact of the Omicron wave on different ethnicities and age groups in the study region. Unfortunately, data on individual comorbidities or health status could not be included and the influence of these factors on hospitalisation rates or length of stay could not be assessed. Our hospitalisation rates do include both hospitalisations *for* and *with* COVID-19 as these were not reported separately during the study period. Our hospitalisation rates per 1,000 reported infections are also likely to be an overestimate due to an under-detection of infections.

### Unanswered questions and future research

This research is a descriptive analysis of the Omicron wave in a region of NZ. While an interaction term between age and ethnicity for hospitalisation rates was observed, the reason for this interaction is currently unclear. It could be due to the effect of age-related vulnerability negating underlying health inequities between Māori, Pacific, and Other ethnicities in younger age groups. It may also reflect a ‘healthy survivor’ effect, whereby Māori and Pacific people who live past 75 years of age are more likely to be in better health. Compared with European and others, the average life expectancy for Māori and Pacific men is 7.6 and 5.6 years shorter, respectively, and 7.4 and 5.5 years shorter for Māori and Pacific women [40].

## Conclusion

Our findings provide evidence for the inequitable impact of the Omicron wave on Māori and Pacific people, through higher per-capita infection and hospitalisation rates. We also confirm that COVID-19 infections have the greatest impact on older people with higher rates of hospitalisation and longer length of stay. Two and three vaccination doses substantially reduce the risk of hospitalisation for all ethnicities and age groups.

## Data Availability

The data used in this research are individual unit records which include COVID-19 infections, hospitalisations, vaccination status, and demographic information. As such, these data are sensitive and were provided by DHBs for the exclusive purpose of the presented research. Furthermore, our ethics approval does not permit sharing of these data. As such, the raw data used to conduct this research are unavailable. Requests for aggregated datasets will be considered in consultation with the DHBs who provided these data, and the University of Waikato Human Research Ethics Committee.

## Supplementary material

For supplementary material accompanying this paper visit https://doi.org/10.1017/S0950268823000572. click here to view supplementary material
