# Supplementary material for: Inequities in COVID-19 Omicron infections and hospitalisations for Māori and Pacific people in Te Manawa Taki Midland region, New Zealand
Source: Epidemiol Infect. 2023 Apr 24;151:e74. doi: 10.1017/S0950268823000572 (PMC10203532; doi:10.1017/S0950268823000572)
Supplement: Supplementary file 1 [file S0950268823000572sup001.docx]

**Supplementary figures and tables**

**
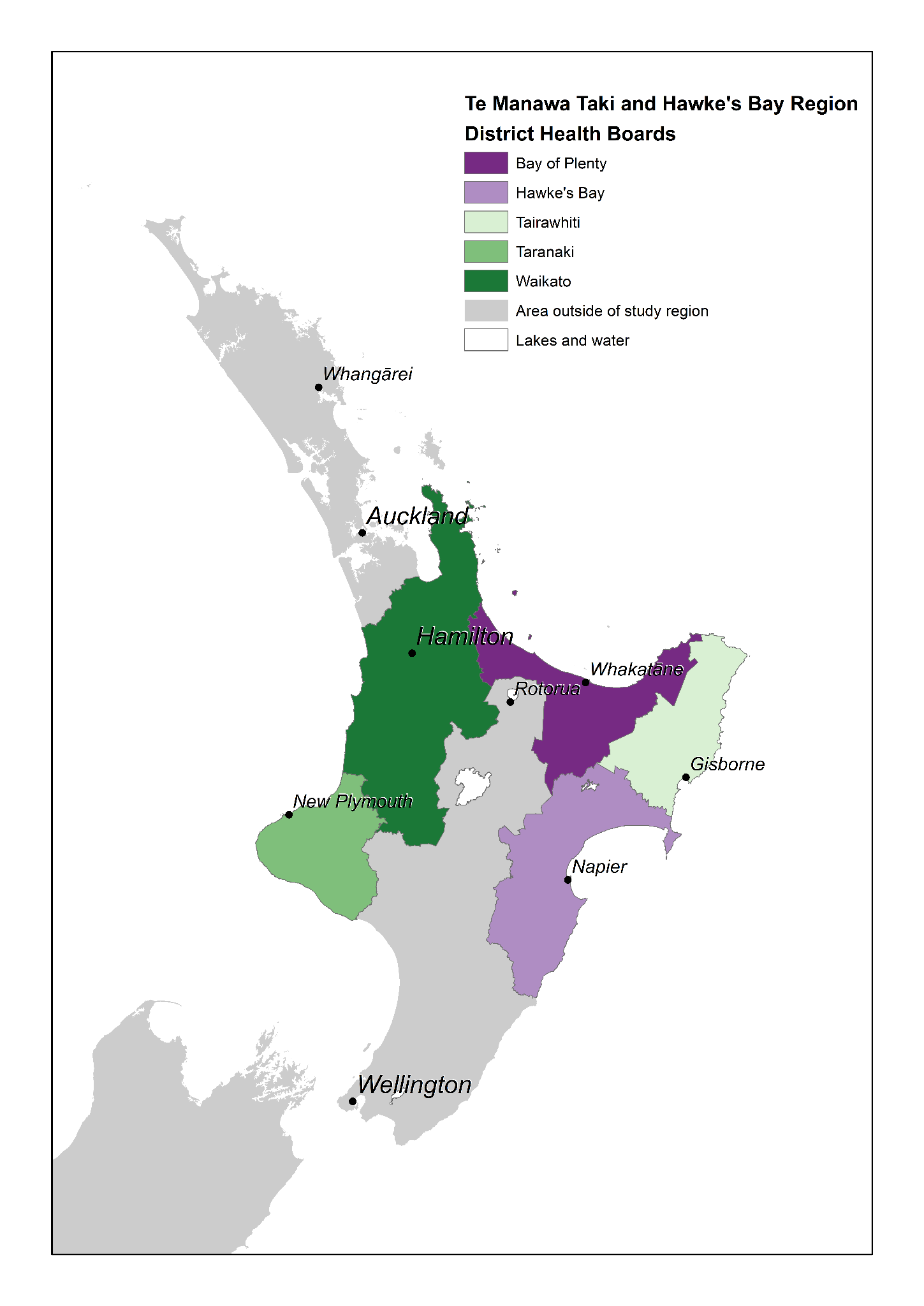
**

**Supplementary Figure 1:** *The District Health Boards included in the study region*

**Supplementary Table 1:** *Fitted additive linear model hospitalisation length of stay (days) by ethnicity, age band and vaccination status for the Bay of Plenty and Waikato DHB regions from 1 Feb - 29 June 2022.*

| **Vaccination status** | **Age band** | **Māori** | **Pacific** | **Other** |
| --- | --- | --- | --- | --- |
| **Unvaccinated** | **0-11** | 0.896 | 1.174 | 1.186 |
|  | **12-24** | 1.818 | 2.096 | 2.108 |
|  | **25-44** | 1.944 | 2.222 | 2.234 |
|  | **45-64** | 4.435 | 4.713 | 4.725 |
|  | **65-74** | 4.707 | 4.985 | 4.997 |
|  | **75+** | 6.885 | 7.163 | 7.175 |
| **Two doses** | **0-11** | 1.271 | 1.549 | 1.561 |
|  | **12-24** | 2.193 | 2.471 | 2.483 |
|  | **25-44** | 2.319 | 2.597 | 2.609 |
|  | **45-64** | 4.81 | 5.088 | 5.1 |
|  | **65-74** | 5.082 | 5.36 | 5.372 |
|  | **75+** | 7.26 | 7.538 | 7.55 |
| **Three doses** | **0-11** | N/A | N/A | N/A |
|  | **12-24** | 1.975 | 2.253 | 2.265 |
|  | **25-44** | 2.101 | 2.379 | 2.39 |
|  | **45-64** | 4.592 | 4.87 | 4.882 |
|  | **65-74** | 4.864 | 5.142 | 5.153 |
|  | **75+** | 7.042 | 7.32 | 7.332 |


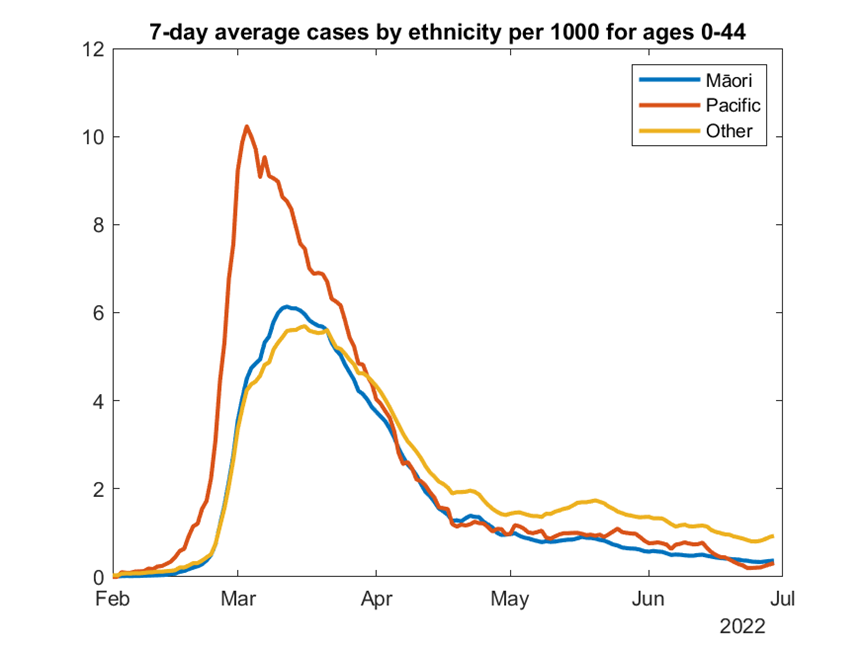


**Supplementary Figure 2:** *7-day average confirmed cases by ethnicity per 1000 for the three youngest age groups (0-44 years).*


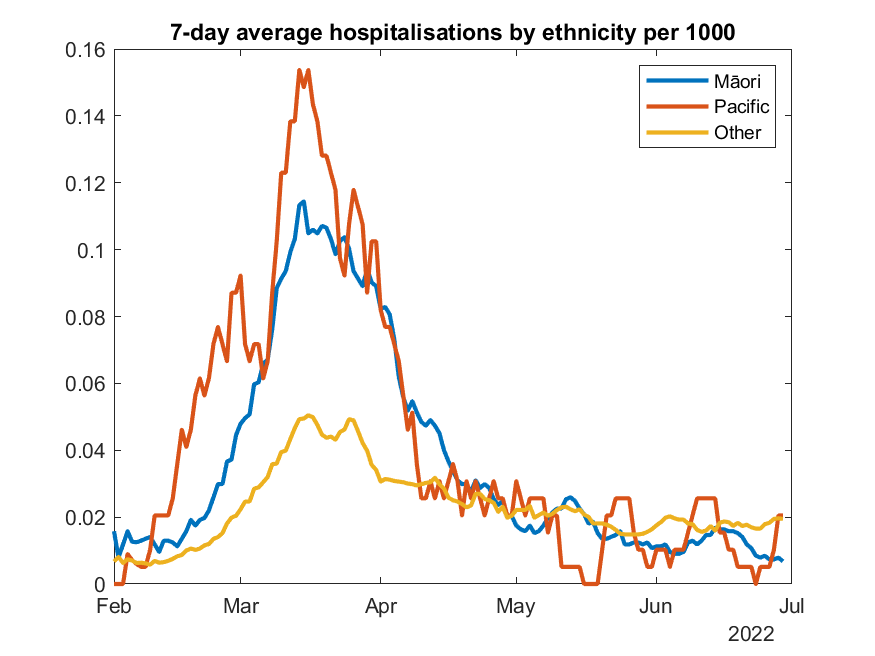


**Supplementary Figure 3**: *7-day average daily admissions for Covid-19 hospitalisations per 1000 individuals by ethnicity for Waikato, Bay of Plenty, Taranaki and Hawkes Bay.*

**Supplementary Table 2:** *Total number of confirmed infections, hospitalisations and the hospitalisation rate per 1000 confirmed infections and per 1000 people by ethnicity, age band and vaccination status for the Bay of Plenty, Taranaki, Hawke’s Bay and Waikato DHB regions combined from 1 Feb – 29 June 2022.*

|  |  | **Confirmed infections** | | | **Total hospitalisations** | | | **Hospitalisations per 1000 confirmed infections** | | | | **Hospitalisations per 1000 people** | | | |
| --- | --- | --- | --- | --- | --- | --- | --- | --- | --- | --- | --- | --- | --- | --- | --- |
| **Vaccination status** | **Age band** | **Māori** | **Pacific** | **Other** | **Māori** | **Pacific** | **Other** | | **Māori** | **Pacific** | **Other** | | **Māori** | **Pacific** | **Other** |
| **Unvaccinated** | **0-11** | 9900 | 1130 | 21338 | 200 | 24 | 250 | | 20.20 | 21.24 | 11.72 | | 3.19 | 3.90 | 2.69 |
|  | **12-24** | 2506 | 231 | 3448 | 71 | 1 | 34 | | 28.33 | 4.33 | 9.86 | | 4.24 | 1.43 | 3.77 |
|  | **25-44** | 2428 | 257 | 3371 | 124 | 12 | 95 | | 51.07 | 46.69 | 28.18 | | 6.19 | 19.26 | 4.26 |
|  | **45-64** | 716 | 72 | 1746 | 83 | 12 | 64 | | 115.92 | 166.67 | 36.66 | | 8.62 | 33.99 | 2.48 |
|  | **65-74** | 141 | 7 | 391 | 43 | 4 | 45 | | 304.96 | 571.43 | 115.09 | | 32.95 | 40.00 | 4.48 |
|  | **75+** | 58 | 7 | 318 | 29 | 3 | 97 | | 500.00 | 428.57 | 305.03 | | 24.49 | 31.25 | 14.39 |
| **Two doses** | **0-11** | 506 | 61 | 2583 | 9 | 2 | 6 | | 17.79 | 32.79 | 2.32 | | 54.22 | 111.11 | 20.07 |
|  | **12-24** | 12783 | 1943 | 25946 | 77 | 10 | 74 | | 6.02 | 5.15 | 2.85 | | 2.10 | 1.99 | 1.01 |
|  | **25-44** | 12456 | 2105 | 25648 | 160 | 26 | 190 | | 12.85 | 12.35 | 7.41 | | 5.19 | 4.79 | 2.32 |
|  | **45-64** | 3962 | 547 | 10250 | 104 | 15 | 109 | | 26.25 | 27.42 | 10.63 | | 6.83 | 8.80 | 2.17 |
|  | **65-74** | 322 | 38 | 1186 | 33 | 4 | 88 | | 102.48 | 105.26 | 74.20 | | 17.39 | 21.62 | 7.77 |
|  | **75+** | 127 | 18 | 689 | 47 | 4 | 126 | | 370.08 | 222.22 | 182.87 | | 66.38 | 51.28 | 19.49 |
| **Three doses** | **0-11** | N/A | N/A | N/A | N/A | N/A | N/A | | N/A | N/A | N/A | | N/A | N/A | N/A |
|  | **12-24** | 2005 | 411 | 7310 | 20 | 0 | 37 | | 9.98 | 0.00 | 5.06 | | 4.90 | 0.00 | 2.08 |
|  | **25-44** | 6604 | 1771 | 31502 | 82 | 19 | 178 | | 12.42 | 10.73 | 5.65 | | 6.11 | 4.38 | 2.25 |
|  | **45-64** | 6966 | 1184 | 31101 | 127 | 19 | 227 | | 18.23 | 16.05 | 7.30 | | 5.49 | 5.97 | 1.81 |
|  | **65-74** | 1633 | 194 | 9823 | 91 | 8 | 240 | | 55.73 | 41.24 | 24.43 | | 10.48 | 8.85 | 3.35 |
|  | **75+** | 660 | 87 | 7629 | 68 | 10 | 725 | | 103.03 | 114.94 | 95.03 | | 18.62 | 24.69 | 11.87 |

**Supplementary Table 3:** *Modelled and observed average length of hospitalisation, by ethnicity, age band and vaccination status for the Bay of Plenty, Taranaki, Hawke’s Bay and Waikato DHB regions combined from 1 Feb – 29 June 2022.*

|  |  | **Modelled hospitalisation average length of stay** | | | **Observed hospitalisation average length of stay** | | |
| --- | --- | --- | --- | --- | --- | --- | --- |
| **Vaccination status** | **Age band** | **Māori** | **Pacific** | **Other** | **Māori** | **Pacific** | **Other** |
| **Unvaccinated** | **0-11** | 0.896 | 1.174 | 1.186 | 1.116 | 1.243 | 1.011 |
|  | **12-24** | 1.818 | 2.096 | 2.108 | 2.058 | 0.930 | 3.528 |
|  | **25-44** | 1.944 | 2.222 | 2.234 | 2.709 | 1.684 | 2.540 |
|  | **45-64** | 4.435 | 4.713 | 4.725 | 3.541 | 3.872 | 2.815 |
|  | **65-74** | 4.707 | 4.985 | 4.997 | 4.566 | 6.655 | 4.842 |
|  | **75+** | 6.885 | 7.163 | 7.175 | 4.610 | 3.363 | 8.203 |
| **Two doses** | **0-11** | 1.271 | 1.549 | 1.561 | 1.689 | 0.528 | 0.303 |
|  | **12-24** | 2.193 | 2.471 | 2.483 | 2.414 | 0.533 | 1.634 |
|  | **25-44** | 2.319 | 2.597 | 2.609 | 2.166 | 1.418 | 2.159 |
|  | **45-64** | 4.81 | 5.088 | 5.1 | 7.142 | 8.065 | 4.394 |
|  | **65-74** | 5.082 | 5.36 | 5.372 | 3.390 | 8.869 | 6.729 |
|  | **75+** | 7.26 | 7.538 | 7.55 | 6.848 | 1.634 | 7.323 |
| **Three doses** | **0-11** | N/A | N/A | N/A | N/A | N/A | N/A |
|  | **12-24** | 1.975 | 2.253 | 2.265 | 0.669 | N/A | 3.132 |
|  | **25-44** | 2.101 | 2.379 | 2.39 | 1.342 | 7.380 | 2.389 |
|  | **45-64** | 4.592 | 4.87 | 4.882 | 4.483 | 3.363 | 4.896 |
|  | **65-74** | 4.864 | 5.142 | 5.153 | 4.947 | 6.678 | 4.775 |
|  | **75+** | 7.042 | 7.32 | 7.332 | 5.632 | 2.178 | 7.612 |

**NB:** Some of the observed cells have very small numbers in them particularly in the elderly age ranges. This is main advantage of considering the modelled average length of stay to smooth out these effects in the small sample sizes.
